# Effective Isolation for Lung Carcinoma Cells Based on Immunomagnetic Separation in a Microfluidic Channel

**DOI:** 10.3390/bios11010023

**Published:** 2021-01-16

**Authors:** Hien Vu-Dinh, Hui Feng, Chun-Ping Jen

**Affiliations:** 1 Department of Mechanical Engineering and Advanced Institute of Manufacturing for High-Tech Innovations, National Chung Cheng University, Chiayi 62102, Taiwan; vuhien260697@alum.ccu.edu.tw; 2 School of Electromechanical and Automotive Engineering, Yantai University, Yantai 264005, China

**Keywords:** aptamer, lung cancer, A549 cells, immunomagnetic

## Abstract

In this paper, we developed an isolation system for A549 human lung carcinoma cells as an effective factor for the early diagnosis of lung cancer. A microfluidic immunomagnetic method was used, in which the combination of immunomagnetic separation and a microfluidic system allowed for increased isolation efficiency with uncomplicated manipulation. In the microfluidic immunomagnetic strategy, A549 cells were combined with aptamer-conjugated carboxylated magnetic beads and then collected in a specified region by applying a magnetic field. The results were recorded using a fluorescence microscope, and the captured targets were then quantified. The isolation efficiency of A549 cells is up to 77.8%. This paper developed a simple working procedure, which is less time consuming, high-throughput, and trustworthy for the isolation of A549 cells. This procedure can be a useful reference method for the development of an effective diagnosis and treatment method for lung cancer in the future.

## 1. Introduction

In recent decades, cancer has been a significant problem in society and the leading cause of human death worldwide. Among the types of cancer, lung cancer accounts for 12.9% of new cases and 19.4% of deaths in total and is consistently leading among common cancers in terms of incidence and mortality [1,2]. Lung cancer has been increasing, especially in women and less developed countries [3]. Therefore, diagnostic solutions that support treatment to prolong the life of patients with lung cancer are essential. The A549 cell line, known as non-small cell lung carcinoma (NSCLC), accounts for 80–85% of all lung cancer cases [4,5]. A549 cells are used as models for the study of lung cancer similar to circulating tumor cells (CTCs) and have been recognized as an effective factor for the early diagnosis of lung cancer [6,7,8].

Several cellular isolation and detection approaches, including electrochemical detection [9], chemiluminescence detection [10], and surface plasmon resonance [11], have been developed to address some of the problems related to the rarity and heterogeneity of CTCs in whole blood samples [12]. Most of these methods include harsh manipulations that cause serious damage to targets. Some systems designed for isolation and capture of cells have also been allowed to thrive in recent years; despite their undeniable benefits, these systems still have limitations. For example, Gou et al. designed a sheathless inertial focusing chip for particle sorting based on the different properties of targets (size) [13]. However, due to the lack of selectivity, the device will face many difficulties in real samples because of the high complexity of the sample with similar physical properties. To increase target selectivity, immunoassay-based methods are usually used via antibody–antigen effective interactions [14]. Some studies have performed the modification of antibodies on surfaces for the subsequent capture and isolation of targets [15,16]. Since the cell size is much larger than antibodies, in reality, the contact area of the cells and the modified surface is limited; therefore, this issue also hinders working efficiency. To overcome the mentioned limitations, immunomagnetic separation (IMS) is a simple solution to these problems; it possesses outstanding advantages such as simple manipulation, high throughput, low cost, and high and reliable detection performance and maintains the viability of CTCs after treatment for subsequent analysis [17,18].

The IMS method is based on a combination of the interaction of cell membrane antigen-specific antibodies and magnetic force. The nano- and microsized magnetic particles used in the IMS method are usually made from superparamagnetic particles covered with a layer of different chemical groups on the surface [19]. The unique magnetic properties of the magnetic beads, their surface-modified ability to target molecules, and large surface-to-volume ratio remarkably increase the isolation yield [19,20]. Many efforts have been devoted to the development of IMS for sample detection with extremely small cell numbers [21]. The combination of IMS and a microfluidic system, which is referred to as the microfluidic immunomagnetic method, has been strongly developed and one of the most popular methods for isolating and detecting CTCs [22] because of the size and shape of the microfluidic channel and the biocompatibility, low sample and reagent consumption, excellent flow control, and high integration of the method [22,23].

Aptamers are short, single-stranded oligonucleotides or peptide molecules; the tertiary structure formation enables the aptamer to bind various targets such as small molecules, bacterial, proteins, and cells. An in vitro process known as Systematic Evolution of Ligands by Exponential enrichment (SELEX) is used for the synthesis of aptamers with high binding affinity and specificity [24]. Due to the features related to binding ability and biocompatibility, the aptamer is becoming increasingly attractive; it has replaced antibodies in many researches [25]. A DNA aptamer specific to the A549 human lung carcinoma cell line was generated by Zhao et al. [26]; the effectiveness of the DNA aptamer has been recognized via numerous subsequent studies [8,27,28]. This aptamer possesses a high affinity for binding to target A549 cells; their high specificity has been investigated and compared to other cancer cell lines and normal lung epithelial cells [26]. Due to the advantages of high thermal stability, low toxicity, as well as high binding affinity for aptamers comparing with antibodies, the aptamer was employed in our study. Moreover, it is also easy to modify molecules (such as SH and NH2) to positions in its structure, which allows it to attach onto the cell’s surface much more easily than antibodies.

**Figure 1:**
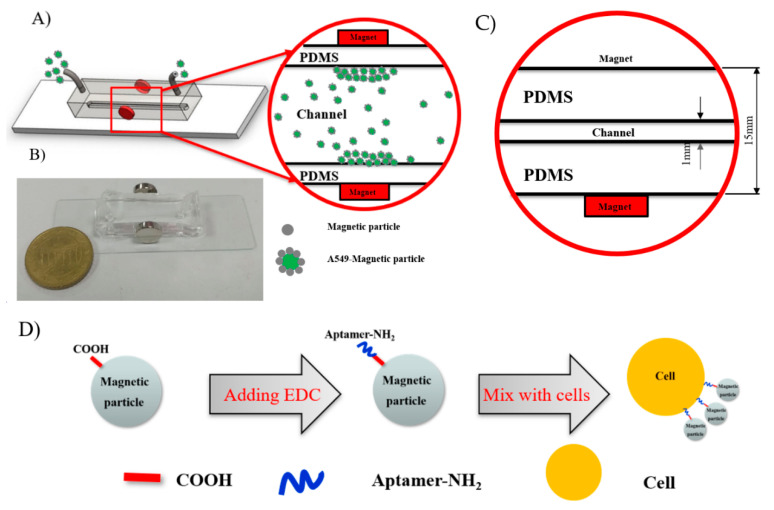
(**A**) Schematic diagram of the proposed chip structure; (**B**) photograph of the proposed chip; (**C**) dimensions of the microchip; (**D**) illustration of the immunomagnetic beads labeling procedure to A549 cells.

In this study, we implemented the microfluidic immunomagnetic method for the isolation of A549 human lung carcinoma cell line. A novel procedure for the efficient and specific association between the A549 cells to the magnetic beads was performed, and then these targets were isolated in a simple structure channel. Carboxylated magnetic beads were coupled to DNA aptamers based on the covalent coupling between carboxylic groups on the bead surface and the DNA aptamer to form immunomagnetic beads. The resultant immunomagnetic beads were anchored on the cell surface because of the specific interaction between the DNA aptamer and target molecule on the cell surface. Then, the microfluidic channel with an external magnetic field was used for the isolation of the functionalized A549 cells (Figure 1A). The results indicated the high isolation performance of the method and facilitated the further development of a new microchannel structure for the effective detection of lung cancer.

## 2. Materials and Methods

### 2.1. Materials

The carboxylated-magnetic beads are superparamagnetic particles with a mean particle diameter of 1.36 μm, concentration of 8.15 mg-Fe/mL, and ligand density of 0.2 mM that were purchased from MagQu Co., Ltd. (New Taipei, Taiwan). The DNA aptamer (5′-ACGCT CGGAT GCCAC TACAG GGTTG CATGC CGTGG GGAGG GGGGT GGGTT TTATA GCGTA CTCAG CTCAT GGACG TGCTG GTGAC-3′-NH_2_), EDC, and NHS were obtained from Sigma-Aldrich (St. Louis, MO, USA). The modification strategy of DNA aptamer on the 3′ end aims to resist nuclease degradation as well as increase the stability of the aptamer [29]. The aptamer used in this study is a full-size aptamer, and it performed satisfactorily for this investigation in our previous study [30]. Calcein green AM and Calcein red-orange AM were bought from Thermo Fisher Scientific (Eugene, OR, USA). Ultrapure water (18.2 MΩ cm at 25 °C) was provided by a Direct-Q system (Milli-Q, Millipore Simplicity, Billerica, MA, USA) for the preparation of buffer solutions. A549 lung adenocarcinoma cells and HeLa cervical cancer cells were cultured in vitro in our laboratory for the experiment.

### 2.2. Microfluidic System Design and Fabrication

Figure 1B shows the texture of the proposed microfluidic system, which consists of a PDMS chamber embedded on a glass substrate with an input and an output connected through a straight microchannel. Two magnets were placed on the center side of the chamber with dimensions of Ø 10 mm × 3 mm. The poles of these two magnets are aligned to each other. The microfluidic device was fabricated by applying the photolithography technique and a casting process.

For the fabrication of a master mold based on the photolithography technique, a silicon wafer was sequentially cleaned with piranha solution (30% H_2_O_2_ and 96% H_2_SO_4_ with a volume ratio of 3:1), acetone, alcohol, and deionized water and dried by nitrogen gas flow. The negative SU-8 photoresist was spin-coated onto the cleaned silicon wafer at 500 rpm for 20 s and 1000 rpm for 35 s to form an SU-8 resist layer with 50 μm thickness. This wafer was then soft-baked at 65 °C for 30 min and 95 °C for 45 min on a programmable hot plate. The wafer was cooled, covered with a microchannel pattern-designed mask (channel width of 1 mm), and exposed under UV light at an exposure dose of 200 mJ/cm^2^ for 95 s. The wafer was then baked again at 65 °C for 15 min and 95 °C for 23 min to complete the polymerization. The master mold was developed by MicroChem’s SU-8 developer, washed with isopropyl alcohol, then hard-baked, and stored for subsequent experiments.

The microfluidic channel was finally obtained by the casting process, which uses a replication procedure of PDMS, including mixing the curing agent, degassing, pouring into the SU-8 mold, baking, piercing, and bonding on a glass substrate. A more detailed procedure of the casting process can be found in our previous research [30]. The height and width of the microfluidic channel were approximately 50 μm and 1 mm, respectively. The parameters of the microfluidic system are shown in Figure 1C.

### 2.3. Theory

In this study, the magnetic bead-labeled A549 cells were considered the oriented targets. The movement trajectory of each magnetic bead-labeled A549 cell in the fluid flow inside the microchannel was determined by the sum of the forces exerted on the cell. According to Newton’s second law, the equation of force acting on each the target is [31]:

(1) $$m_{c}\frac{du_{c}}{dt}=F_{m}+F_{g}+F_{d}+F_{B}+F_{L}$$

where $m_{c}$ and $u_{c}$ are the mass and velocity of the target (magnetic bead-labeled A549 cell), respectively, and $F_{m}$, $F_{d}$, $F_{g}$, $F_{B}$, and $F_{L}$ are the magnetic force, drag force, gravity force, Brownian force, and lift force, respectively. In practice, a high magnetic field is regularly applied; therefore, magnetic force and drag force are the two dominant forces and the remaining forces can be ignored. The equations of magnetic force acting on an immunomagnetic bead with permeability constant of vacuum μ_0_ and the drag force acting on a captured target can be expressed as [17]:

(2) $$F_{m}=\frac{V_{c}\left(\chi_{p}-\chi_{f}\right)}{\mu_{0}}\left(B\cdot \nabla \right)B$$

(3) $$F_{d}=3\pi \eta d_{c}\left(u_{f}-u_{c}\right)$$

where $V_{c}$, $\chi_{p}$, and $\chi_{f}$ are the volume and magnetic susceptibilities of the immunomagnetic bead, respectively; η and $u_{f}$ are the dynamic viscosity and velocity of the fluids, respectively; $d_{c}$ and $u_{c}$ are the diameter and velocity of the captured target, respectively; B is the magnetic field. In areas with high magnetic density (where the permanent magnets were applied), the magnetic force was increased and the net force of the target was changed; thus, the targets moved from the flow direction to the location of a large magnetic field gradient. Therefore, the targets can be oriented to a specified location in the microchannel.

### 2.4. Magnetic Bead Conjugation

Carboxylated magnetic beads (10 μL, ligand density of 0.2 mM) were cleaned three times with PBS washing buffer solution (PBS1x, pH 7.4) in a tube by concentrating the magnetic beads with a magnet bar and then extracting the supernatant. The carboxylic groups on the magnetic beads were activated by 40 μL of EDC/NHS solution (0.05 M NHS and 0.25 M EDC in 0.1 M MES buffer) for 30 min. The amine-labeled aptamers were diluted in a binding buffer solution (0.1 mg tRNA and 1 mg BSA in 1 mL of the washing buffer) to a concentration of 20 μM, then heated at 95 °C for 5 min, and cooled at room temperature for 15 min. After removing the unreacted EDC/NHS in the carboxylic group activation step by the washing buffer solution, 25 μL of the post-treatment aptamer was added to the tube containing the activated-magnetic beads and then incubated for 90 min to bind the aptamers to the magnetic beads. The reaction with the DNA aptamer was performed immediately to prevent the hydrolysis of the amine-reactive esters. Finally, the resultant immunomagnetic beads were preserved in1xPBS buffer.

### 2.5. Isolation of A549 Cells

**Figure 2:**
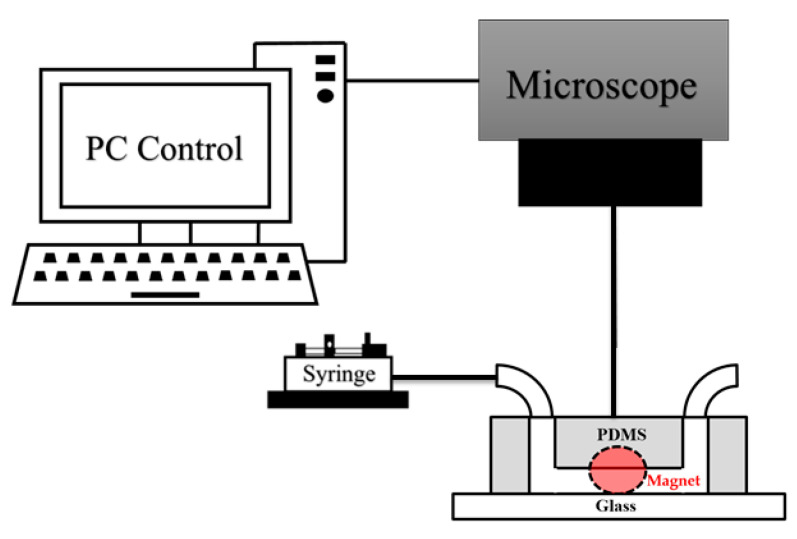
Experimental setup of the isolation system.

A549 and HeLa cell samples were stained with Calcein green AM and Calcein red-orange AM for 15 min, respectively. The resultant samples were incubated with the immunomagnetic beads for 15 min. Finally, the obtained experiment sample was injected into the microfluidic channel by a syringe pump (Model KDS 101, KD Scientific Inc., Holliston, MA, USA), and the results were recorded through a fluorescence microscope (BX43, Olympus, Tokyo, Japan). The microfluidic channel was also filled with the washing buffer solution for 30 min before injecting the experiment sample to minimize surface absorption. The experimental setup is shown in Figure 2.

## 3. Results and Discussion

### 3.1. Characteristics of Functionalized A549 Cells

The functionalization of aptamers or antibodies on a carboxylated magnetic bead was carried out based on intermediate carbodiimide reaction, which has been widely implemented in many previous studies and showed high stability as well and strong binding capacity [17,21]. *N*-(3-Dimethylaminopropyl)-*N*′-ethylcarbodiimide hydrochloride (EDC)/*N*-hydroxysuccinimide (NHS) solution was used in the experiment to prepare amine-reactive esters derived from carboxylate groups to react with the amino group of the DNA aptamer. Although NHS is not required for the carbodiimide reaction, the presence of NHS as a catalyst enhances coupling efficiency considerably. The NHS esters were hydrolyzed over time; thus, we investigated the optimal time for activation reaction with the EDC/NHS solution. Results showed that a duration of 30 min for the given reagent concentrations is ideal for the subsequent modification step of the aptamers. The process of binding between the magnetic beads and A549 cells is illustrated in Figure 1D.

**Figure 3:**
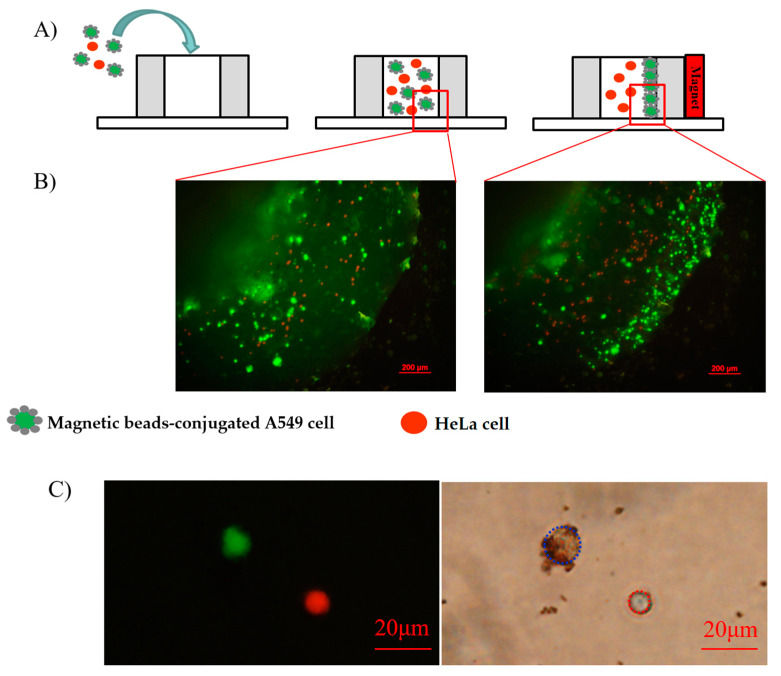
Characterization of the functionalized A549 cells. (**A**) Schematic illustration of the evaluation experiment for the combination of immunomagnetic beads and A549 cells. (**B**) Fluorescent images of the A549 cells and HeLa cells before and after applying a permanent magnet. (**C**) Fluorescence image and conventional image of the treated A549 cell and the stained HeLa cell.

A circular well was designed using a circular hollow structure made of polydimethylsiloxane (PDMS) placed on a glass substrate with pool diameter and height of 4.5 and 6.5 mm, respectively, to assess the effectiveness of the modification of the immunomagnetic beads and their ability to bind to the cell surface. An amount of 100 μL of the sample experiment was prepared by the incubation of the mixture of Calcein green AM-stained A549 cells and Calcein red-orange-stained HeLa cells to form the immunomagnetic beads as mentioned. The concentrations of the A549 cells and HeLa cells are both 1 × 10^5^ cells/mL. This sample was put into the well through a pipette; a permanent magnet was then applied to one side of the well (Figure 3A). The images observed under a fluorescence microscope with an excitation wavelength of 490 nm showed the isolation of A549 cells in the well obviously. The A549 cells transported to one side of the well, whereas the HeLa cells seemed immobile. The magnetic force generated by the permanent magnet attracts magnetic beads on the cell surface to a high-magnetic field gradient position; therefore, the A549 cells were transported to the side with the permanent magnet, as shown in Figure 3B.

In addition, the fluorescence and conventional images of the HeLa cells and labeled A549 cells were also recorded and investigated. Figure 3C shows the A549 cells with green fluorescence and the HeLa cells with red fluorescence. The conventional image indicates that the magnetic beads were coated on the A549 cells effectively, whereas the magnetic beads and HeLa cells had no interaction. In addition, in previous research, Zhao et al. also tested the specificity of this DNA aptamer on a variety of subjects such as other cancer cell lines (MCF7) and normal lung epithelial cells (HPL1 cell line) [26]. These results showed the excellent binding of the magnetic beads to A549 cells and the high specificity of the DNA aptamer.

### 3.2. Effective Isolation of A549 Cells

**Figure 4:**
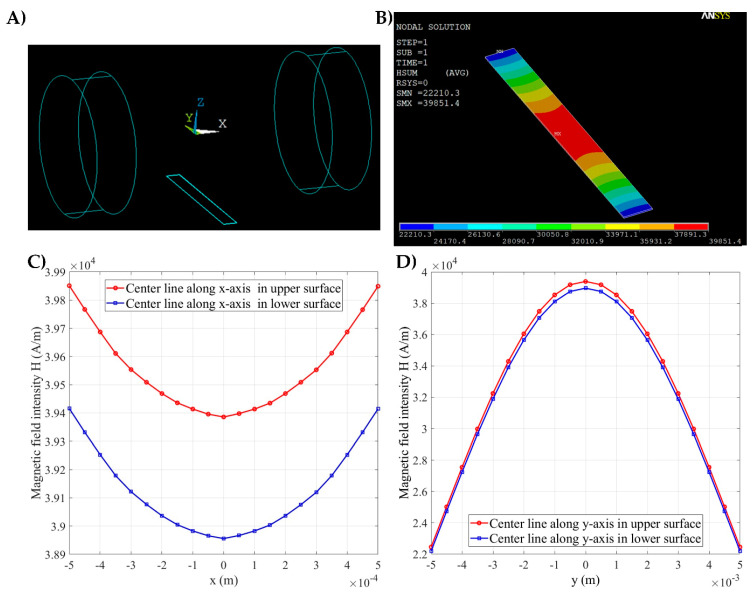
The ANSYS Emag software for the simulation of the magnetic field in the proposed system. (**A**) Model of permanent magnet and microfluidic channel. (**B**) Simulation of the magnetic field intensity distribution in the microfluidic channel. (**C**) Magnetic field intensity values on the centerline of the upper and lower surfaces of the flow channel along the *x*-axis. (**D**) Magnetic field intensity values on the centerline of the upper and lower surfaces of the flow channel along the *y*-axis.

**Figure 5:**
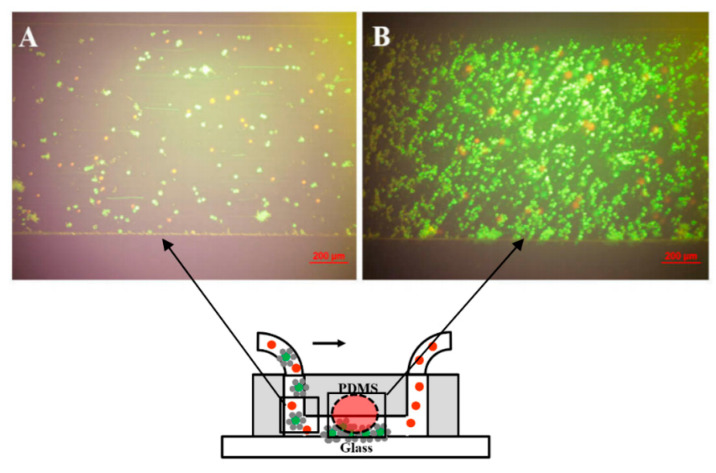
Fluorescent images of the A549 cells and HeLa cells at different positions in the microfluidic channel of the isolation system. (**A**) Image of non-magnet position. (**B**) Image of the position applied permanent magnet.

An amount of 1 mL of the experiment sample with a cell concentration of 1 × 10^5^ cells/mL was prepared. A flow rate of 10 μL/min was used in our study. The experiment was then carried out in accordance with the setup that was mentioned. Magnetic field was generated by applying two magnets on the center of each side of the PDMS chambers. The intensity distribution of the magnetic field was also calculated via the ANSYS Emag software (Figure 4). The model of the permanent magnet and microfluidic channel is shown in Figure 4A. The variation in the magnetic field intensity on the 1 mm width of the microchannel along the *x*-axis was 0.05 × 10^4^ A/m, as shown in Figure 4C. This variation value is relatively small compared with the average variation value; therefore, the magnetic field distribution along the *x*-axis can be considered uniform (Figure 4B). Figure 4B also indicates that the distribution of the A549 cells captured in the magnetic area along the *x*-axis is almost uniform without obvious concentration at the two microchannel boundaries. Figure 4D presents the magnetic field intensity values on the centerline of the upper and lower surfaces of the flow channel along the *y*-axis. Figure 4D shows the decrease in magnetic field intensity from the center to the ends of the microchannel along the *y*-axis. Therefore, the A549 cells were captured at the center of the microfluidic channel (magnetic area), whereas the HeLa cells were swept along the fluidic flow and washed away to the outlet of the microchannel. Figure 5A shows the distribution of A549 and HeLa cells in the fluidic flow in the non-magnetic area. The results show that the concentration of A549 cells in the non-magnetic area was substantially less than that in the magnetic area (Figure 5B). According to experimental results, A549 cells can be captured under the magnetic field generated by the magnet. The simple simulation could provide approximately the required strength of the magnetic field to cause the cells labeled with magnetic beads to move.

Flow rate investigation was performed by injecting the prepared sample into the microfluidic system and isolating the target at different flow rates (10, 20, 30, and 35 μL/min). After isolation, the number of captured A549 cells was measured by calculating the amount of A549 cells at the output via ImageJ software. The experiment was repeated 5 times for each flow rate; the average value was recorded for drawing the graphs. The cell capture efficiency was defined as in Equation (4):

(4) $$\mathrm{Capture}\;\mathrm{efficiency}\;=\frac{\mathrm{The}\;\mathrm{number}\;\mathrm{of}\;A549\;\mathrm{cells}\;\mathrm{captured}\;\mathrm{in}\;\mathrm{the}\;\mathrm{channel}\;}{\mathrm{The}\;\mathrm{total}\;\mathrm{number}\;\mathrm{of}\;A549\;\mathrm{cells}\;\mathrm{injected}\;\mathrm{into}\;\mathrm{the}\;\mathrm{channel}}$$

**Figure 6:**
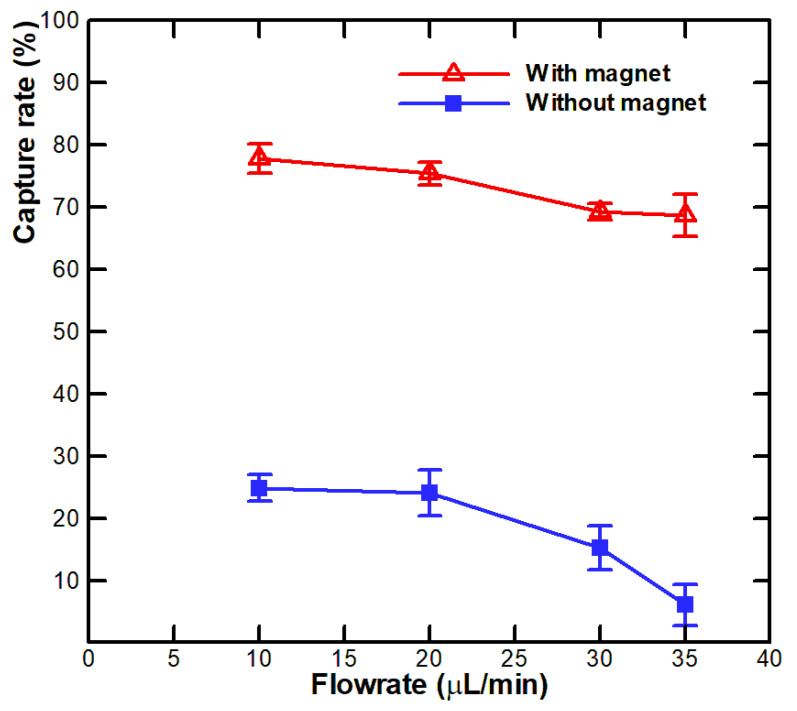
Isolation results of the A549 cells at different flow rates.

Figure 6 indicates the results with the designated flow rates. At the flow rates of 10, 20, 30, and 35 μL/min, the capture rates were 77.8, 75.4, 69.2, and 68.7%, respectively. The flow rate of 10 μL/min showed the best capture capacity of up to 77.8 ± 2.4% and retained a negligible amount of HeLa cells in the microfluidic channel. The capture rate of HeLa cells was less than 1% at the implemented flow rates. The phenomena also indicate that the trapping on the surface of the channel is negligible for biological cells with small gravity force (those without binding to magnetic beads), such as HeLa cells. Moreover, the captured A549 cells were released from the channel at the flow rate larger than 40 μL/min while the magnets were removed. We conducted experiments with the same cell concentration at the rate of 10 μL/min to test the repeatability of the method. We found that the method has high stability. The slope of the graph when using the magnet and without the magnet also shows the influence of the flow rate to the capture ability. Capture rates were less affected for the cases when the magnet was applied, but there was a rapid decline in the capture efficiency at high flow rates when not applying the magnet. These results clearly show that the proposed system can isolate A549 cells effectively and with high specificity. The isolation efficiency is comparable to some of the previous systems for cell isolating, for example, in the study by Kwak et al. [32], 3 mL of 1 × 10^5^ cells/mL (3 × 10^5^ cells) was isolated. Most of the target circulating tumor cells (CTCs) were isolated in the system, of which 71.3% of isolated target CTCs were isolated in the first half of the area of the system, while 76.9% of non-target CTCs were collected at the latter half of the area. The isolating efficiency of the system in our current study is comparable to the published report in literature; moreover, the proposed system has the advantages of being easy to manufacture, low cost, as well as simple in operation and integration.

## 4. Conclusions

In summary, we successfully developed an isolation system for human lung carcinoma cell line (A549) based on the microfluidic immunomagnetic method. This system has a simple working procedure, is less time consuming, has high throughput, and is trustworthy. The isolation efficiency for A549 cells is up to 77.8%. The effective binding of the magnetic beads on the surface of A549 cells opens up many possible applications for the future detection of A549 cells. The development of a new microfluidic channel structure for the collection and detection of A549 cells is feasible herein. The main purpose of this study is to demonstrate the feasibility and efficiency of this approach. Detailed analysis of the experimental parameters, such as the concentration of cells, the composition of the sample or the buffer solution, and the size of magnetic beads as well as the number of aptamers binding on the surfaces of beads, could be conducted in future work. Therefore, we expect that this study will be seen as a useful reference method for the development of an effective diagnosis and treatment method for lung cancer in the future.

## Data Availability

The data presented in this study are available on request from the corresponding author.
